# Identification of GASA Protein Family Expression Levels in Cotton (*Gossypium hirsutum* L.) Affected by *Verticillium wilt*

**DOI:** 10.3390/biology15161434

**Published:** 2026-08-20

**Authors:** Cong-Hua Feng, Yi Liu, Suen Liu, Junyi Geng, Hui Sun, Hongwei Cao, Ruixuan Liu, Yuyuan Qian, Baosheng Guo

**Affiliations:** 1 Institute of Cotton, Hebei Academy of Agriculture and Forestry Sciences, Key Laboratory of Cotton Biology and Genetic Breeding in Huanghuaihai Semiarid Area, Ministry of Agriculture and Rural Affairs, Hebei Key Laboratory of Cotton Bio-Breeding and Cultivation Physiology, Shijiazhuang 050051, China; 2 China Grain Reserves Group Ltd. Company, Beijing 100089, China

**Keywords:** *GhGASA*, cotton, disease resistance, gene family, biotic stress, transcriptional regulation

## Abstract

*Verticillium wilt*, caused by *Verticillium dahliae*, is a major constraint on cotton production. The GASA (Gibberellic Acid-Stimulated in *Arabidopsis*) proteins are cysteine-rich peptides induced by gibberellin signaling; however, their systemic role in upland cotton (*Gossypium hirsutum*) resistance to *Verticillium wilt* remains elusive. Here, we identified 40 *GhGASA* members from the *G. hirsutum* genome, which were clustered into three subfamilies. All members possessed a typical gibberellin-regulated domain, and segmental duplication was the primary driver of family expansion. Transcriptomic analyses revealed tissue-specific expression, with high abundance in pistils and roots, and distinct responsive patterns to salt, drought, and cold stresses. Quantitative real-time PCR (qRT-PCR) showed that multiple *GhGASA* genes were significantly upregulated following *V. dahliae* inoculation, with transcript levels being much higher in roots and stems than in leaves. Notably, *GhGASA17*, harboring ethylene-responsive elements, was identified as a key candidate involved in the defense response. Overall, this study provides a comprehensive characterization of the *GhGASA* gene family, offering a theoretical foundation for understanding its regulatory functions in *Verticillium wilt* resistance and delivering potential genetic resources for molecular breeding in cotton.

## Simple Summary

Cotton is one of the most important crops worldwide, but its production is severely threatened by Verticillium wilt, a soil-borne disease that causes wilting and death of the plants, leading to major yield losses. In this study, we investigated a group of genes known as the GASA family, which produce small proteins involved in plant growth, stress responses, and defense. We identified 40 GASA genes in upland cotton and examined their behavior under different stress conditions, especially Verticillium wilt. Our results showed that several of these genes became more active when the cotton plants were infected, particularly in the roots and stems where the disease attacks first. One gene in particular, GhGASA17, showed a very strong response, suggesting it may play an important role in helping cotton resist the disease. This work provides new insights into how cotton defends itself against Verticillium wilt and offers potential genetic resources that could be used in breeding programs to develop more resistant cotton varieties. These findings may ultimately help reduce crop losses and improve the sustainability of cotton production.

## 1. Introduction

Cotton is the world’s most important source of natural textile fibers and a key strategic crop for both the agricultural economy and as an industrial raw material [1]. Its seeds serve as raw material for oil extraction and as a high-quality protein feed, while the entire cotton plant holds significant potential for applications in bioenergy and other fields, thereby occupying a vital position in global agriculture [2,3]. However, cotton yield and fiber quality are highly susceptible to severe impacts from biotic stresses such as *Verticillium wilt* [4]. *Verticillium wilt* is a soil-borne vascular disease caused by *Verticillium dahliae* [5,6]. Once the pathogen infects the plant, it blocks the xylem vessels, leading to leaf wilting, yellowing, and even plant death [7,8]. In particular, infection during the flowering and boll-forming stages severely impedes the transport of photosynthetic products to the cotton bolls, resulting in massive boll drop, poor fiber development, and a sharp decline in yield [9,10]. Therefore, a thorough analysis of the molecular mechanisms underlying cotton resistance to *Verticillium wilt* and the identification of key resistance genes are of great significance for ensuring the sustainability of global cotton production.

In recent years, significant progress has been made in understanding the molecular mechanisms underlying the interaction between cotton and *Verticillium dahliae* [10,11]. At the effector level, several secreted proteins have been shown to suppress plant immunity or activate defense responses [12,13]. For example, *VdEPD1* is recognized by the cotton cytoplasmic kinase *GbEIR5A*, triggering effector-triggered immunity and enhancing disease resistance [14]; *VdEPG1* interacts with *GhSTH-2*, disrupting host jasmonic acid signaling and ROS homeostasis [15]; and *Vd06254* targets *GhMYC3*, disrupting hormonal synergy between jasmonic acid and strigolactones [16]. In plant immune signaling, the MAPK cascade serves as a central regulatory hub for cotton resistance [17]. Furthermore, key enzymes such as ethylene synthase *GhACS2/6* and glutamine synthetase *GhGLN1.5* have been shown to positively regulate cotton resistance through different metabolic pathways [18,19,20,21]. These studies have systematically constructed a multilevel regulatory network of cotton immune signaling.

Over the course of their long evolutionary history, plants have developed a large number of small-molecule cysteine-rich proteins (CRPs), which act as signaling molecules or defense peptides and play key regulatory roles in plant growth, development, and environmental adaptation [22,23,24]. GASA (Gibberellic Acid-Stimulated in *Arabidopsis*) proteins represent an important class of CRPs; they are named for their initial identification in *Arabidopsis* as being induced by gibberellic acid (GA) signaling [25,26]. Since the discovery of the first *GASA* family member, *GAST1*, in tomato, this family has been extensively identified and reported in various plants. In the model plant *Arabidopsis*, 15 *GASA* members have been characterized, with *AtGASA4* and *AtGASA6* shown to promote GA responses and exhibit redox activity [27], while *AtGASA1* and *AtGASA4* positively regulate plant resistance to *Pseudomonas syringae* through the SA pathway [28]. In rice, *OsGSR1* has been demonstrated to participate in the crosstalk between gibberellin and brassinosteroid signaling pathways [29]. In *Populus*, genome-wide analysis identified 31 *GASA* genes with differential responses to various phytohormones and stress treatments [30,31]. In *Medicago truncatula*, 31 *GASA* genes were identified and shown to respond to ABA, salt, and drought stress [32]. In *Hevea brasiliensis*, comprehensive transcriptional analysis revealed that *HbGASA* genes respond to *Colletotrichum gloeosporioides* infection and are involved in ROS production, a key early event in plant innate immunity [33,34]. More recently, *GASA* family members have also been identified in mangrove plants [35]. Studies have found that the expression of *GASA* genes is regulated by various plant hormones, including GA, ABA, JA, and SA [26,36], revealing the central role of GASA proteins in integrating multiple hormonal signals to coordinate defense responses. Overall, the *GASA* gene family plays a crucial positive regulatory role in plant disease resistance through various mechanisms, including the production of ROS, participation in crosstalk between hormone signaling pathways, and direct action as antimicrobial peptides. However, to date, systematic studies on the role of the *GASA* gene family in *G. hirsutum* under *Verticillium wilt* stress remain lacking.

Previous transcriptomic analyses by our research group indicated that the expression level of *GhGASAs* in the *Verticillium wilt*-resistant variety *Ji631* was significantly higher than that in the wild-type *TM-1*. To investigate the role of the *GhGASA* gene in *Verticillium wilt* resistance in *G. hirsutum*, this study conducted a genome-wide identification and functional annotation of the *GhGASA* family, providing a theoretical basis for elucidating its regulatory functions in stress responses and growth and development. A total of 40 *GhGASA* candidate genes were identified, and predictive analyses were conducted on their chromosomal localization, gene amplification, gene structure, and promoter cis-acting elements. Additionally, the physicochemical properties, subcellular localization, motifs, and phylogenetic relationships of the encoded proteins were predicted. For *GhGASA* members associated with *Verticillium wilt* stress, the expression patterns of the representative gene *GhGASA* were analyzed in detail. This study, for the first time, comprehensively employed genomics, transcriptomics, and qRT-PCR technologies to screen for *GhGASA17* as a promising candidate gene. The findings will provide new insights and methodologies for cotton resistance research against *Verticillium wilt* and serve as a reference for disease resistance studies in other crops.

## 2. Materials and Methods

### 2.1. Test Materials

The *Verticillium dahliae* strain used in this study was *Vd991*, which is preserved at the Cotton Research Institute of the Hebei Academy of Agricultural and Forestry Sciences. This strain was inoculated onto potato-glucose agar (PDA) medium and incubated for 7 days at 25 °C in the dark. *Vd991* was inoculated into 200 mL of Czapek liquid medium and then cultured on a shaking incubator at 24 °C and 200 rpm for 5 days. The resulting conidia were collected, filtered through a sterile filter membrane, and the spore concentration was adjusted to 1 × 10^7^ spores/mL using sterile ddH_2_O in a hemocytometer. Cotton seeds (*Gossypium hirsutum* L. cv. *JIN668*) were surface-sterilized, germinated, and grown in vermiculite under greenhouse conditions (28 °C day/25 °C night, 16-h light/8-h dark photoperiod) until the four-leaf stage (approximately 28 days post-germination) [37]. Seedlings of uniform size were selected for inoculation. The roots were gently washed with sterile water to remove vermiculite residues and then immersed in the conidial suspension (1 × 10^7^ conidia/mL) for 40 min. Control plants were immersed in sterile distilled water under the same conditions. After inoculation, all seedlings were transplanted into pots containing sterilized soil and maintained under the same greenhouse conditions. The experiment was conducted with three independent biological replicates, each containing 12 seedlings per treatment (36 seedlings in total per treatment). Root, stem, and leaf tissues were collected at 0, 4, 12, 24, and 72 h and 6 days post-inoculation (dpi) to capture both early and late transcriptional responses to *V. dahliae* infection. The sampling time points were designed based on preliminary disease development observations and previous studies on cotton-*Verticillium* interaction. All collected samples were immediately frozen in liquid nitrogen and stored at −80 °C until RNA extraction.

### 2.2. Identification of the GhGASA Gene Family in G. hirsutum and Analysis of Their Protein Properties

The *G. hirsutum TM-1* (version v2.1) genome, CDS, amino acid sequences, and the 2000 nucleotides upstream of the ATG promoter were extracted from the COTTONOMICS database (http://cotton.zju.edu.cn/, accessed on 4 August 2025) [38]. Members of the *GhGASA* gene family were identified by searching the reference genome using the Gibberellin-regulated protein Hidden Markov Model (PF02704) provided by the Interpro database (https://www.ebi.ac.uk/interpro/, accessed on 10 August 2025). *GhGASAs* were named according to their chromosomal location (*GhGASA1*-*GhGASA40*). *GhGASA* gene locations and chromosome sizes were obtained from the COTTONOMICS database and visualized using TBtools-II (version x64_2_102) software [39]. Protein physicochemical property predictions were performed via the ExPASy website (https://www.expasy.org/, accessed on 21 August 2025). Subcellular localization predictions for the *GhGASA* gene family were performed using the PSI (Plant Subcellular Localization Integrative Predictor) website (http://bis.zju.edu.cn/psi/, accessed on 28 August 2025).

### 2.3. Sequence Alignment and Phylogenetic Analysis of the GhGASA Gene Family

A phylogenetic tree of *GhGASAs* was constructed using the MEGA 12.1.2 parsimony method, and branch reliability was assessed via 1000 bootstrap repetitions [40]. The resulting tree file was visualized using Adobe Illustrator (version 2019) to clearly illustrate the evolutionary relationships.

The amino acid sequences of *GhGASA17*, *GhGASA38*, *AtGASA14*, *CrGASA4* from the NCBI database (https://www.ncbi.nlm.nih.gov/, accessed on 7 October 2025). Sequence alignment was performed using the online tools ClustalW and EDScript/ESPrip (https://www.genome.jp/tools-bin/clustalw; https://espript.ibcp.fr/ESPript/ESPript/index.php, accessed on 18 October 2025), and the results were plotted; simultaneously, their protein tertiary structures were predicted using SWISS-MODEL (https://swissmodel.expasy.org/, accessed on 18 October 2025) [41].

### 2.4. Structural and Evolutionary Analyses of the GhGASA Gene Family

The exon–intron structures of the *GhGASA* genes were visualized using TBtools based on the gene IDs and the GFF3 annotation file. Protein sequences were analyzed using the MEME Suite (default parameters, motif count = 5) to identify conserved motifs, and were submitted to the NCBI Conserved Domain Database for domain annotation [42]. By integrating these data, we used TBtools to jointly construct phylogenetic trees, motif maps, and gene structure diagrams, thereby systematically characterizing the sequence, evolutionary, and structural features of the *GhGASA* family [43].

MCScanX analysis was performed on the FASTA and GFF3 files of the *G. hirsutum* genome using the default TBtools parameters to identify homologous genes and assess collinearity with other species [44]. This yielded three files: GFF, CTL, and Collinearity. Unnamed chloroplast and mitochondrial sequences were manually removed from the CTL file, which was then reordered and finally visualized using TBtools [45].

Cis-acting elements in the 2000-bp sequence upstream of the *GhGASA* gene family promoter were predicted using the PlantCARE website (https://bioinformatics.psb.ugent.be/webtools/plantcare/html/#opennewwindow, accessed on 21 October 2025), and the results were visualized [46].

### 2.5. Analysis of Transcriptional Expression in the GhGASA Gene Family

To characterize the expression profiles of the *GhGASA* genes, we retrieved the FPKM expression matrices for eight cotton tissues (root, stem, leaf, etc.) and three abiotic stress treatments (salt, simulated drought, and cold) from the COTTONOMICS database (http://cotton.zju.edu.cn/10.rnasearch.html, accessed on 4 December 2025) [38]. Heatmaps were generated using the HeatMap module in TBtools (with row-scale normalization and default settings for all other parameters) [39] to visualize the expression patterns across tissues and under different stress conditions. The transcriptomic datasets used in this study correspond to the RNA-seq data of *G. hirsutum TM-1* generated by the COTTONOMICS project [38]. These datasets are publicly accessible under the accession number CNP0002656. These datasets were selected because they represent the most comprehensive and standardized transcriptomic resources currently available for *G. hirsutum TM-1*, covering both developmental stages and multiple stress conditions in a uniform genetic background, which allows for systematic comparison of *GhGASA* expression patterns. Total RNA was extracted from roots, stems, and leaves of four-leaf-stage cotton seedlings (approximately 28 days post-germination) following *V. dahliae* root inoculation using the RNA Simple kit (DP419, Tiangen Biotech; Beijing; China) and reverse transcribed using the FastKing cDNA Synthesis Kit (KR116, Tiangen Biotech; Beijing; China). Specific primers were designed using PrimerQuest (IDT) (Supplementary Table S5). qRT-PCR was performed using 5×SNP Genotyping Lyo qPCR MIX (CW3398S, Beijing ComWin Biotech Co. Ltd., Beijing, China) on a CFX96 Touch™ system (Bio-Rad, Hercules, CA, USA), following the reaction protocol previously described in our laboratory.

### 2.6. Statistical Analysis

We analyzed the qRT-PCR experimental data using SPSS v. 31.0 (SPSS, Inc., Chicago, IL, USA). For relative gene expression, we performed one-way analysis of variance (ANOVA) with LSD multiple comparison tests (*p* < 0.05). Prior to performing the ANOVA, we tested the data for normality and homogeneity of variances [47].

## 3. Results

### 3.1. Identification of the GhGASA Gene Family and the Physicochemical Properties of Its Proteins

To systematically elucidate the role of the *GhGASA* gene family in cotton’s resistance to *Verticillium wilt*, we identified 40 members of the *GhGASA* gene family (Supplementary Table S1). Analysis of the physicochemical properties of the family proteins revealed that *GhGASAs* are small proteins, encoding 76–264 amino acids (aa) on average (112.6 aa); their molecular weights are generally less than 30 kDa. The isoelectric point (pI) ranged from 6.04 to 9.99, with 2 acidic proteins and 38 basic proteins. Eleven members have an instability index below 40, indicating high stability; twenty-nine protein members have a Grand Average of Hydropathicity (GAH) below 0, indicating hydrophilicity, while the remaining 11 members are hydrophobic. Subcellular localization predictions show that the vast majority of GhGASA proteins are localized to the Golgi apparatus and the nucleus.

**Figure 1:**
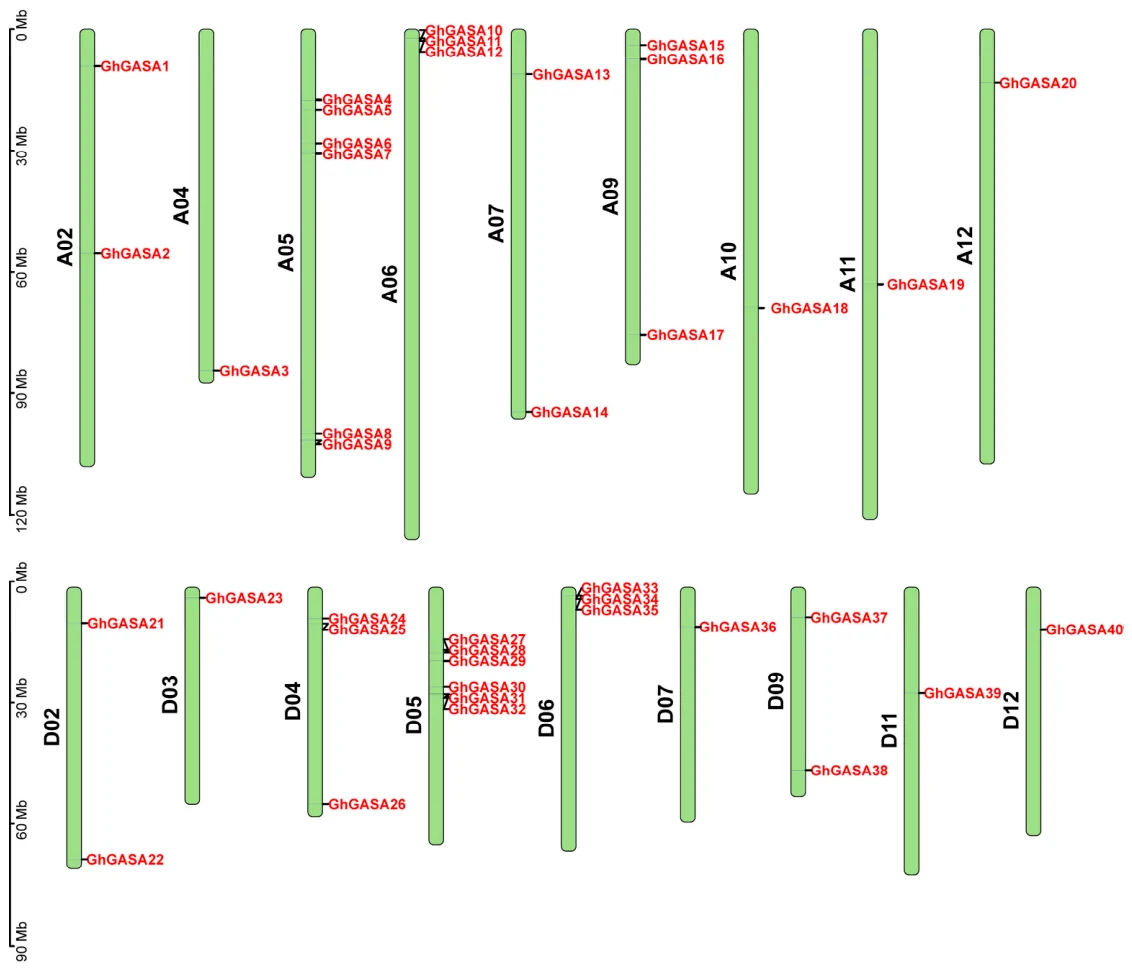
Mapping of 40 *GhGASA* genes on the cotton chromosome. The left side shows the chromosome length, with the scale in megabases. The black chromosome numbers are displayed to the left of each chromosome. The gene names on the right side of the chromosome correspond to the locations of the *GhGASA* genes.

Chromosomal localization shows that the *GhGASA* gene family is distributed across 18 chromosomes, with chromosome D05 hosting the largest number of members (6). The *GhGASA* gene family contains four tandem duplication gene pairs (*GhGASA10/11*; *GhGASA27/28*; *GhGASA31/32*; *GhGASA33/34*) (Figure 1).

### 3.2. Phylogenetic Analysis and Sequence Alignment of the GhGASA Gene Family

**Figure 2:**
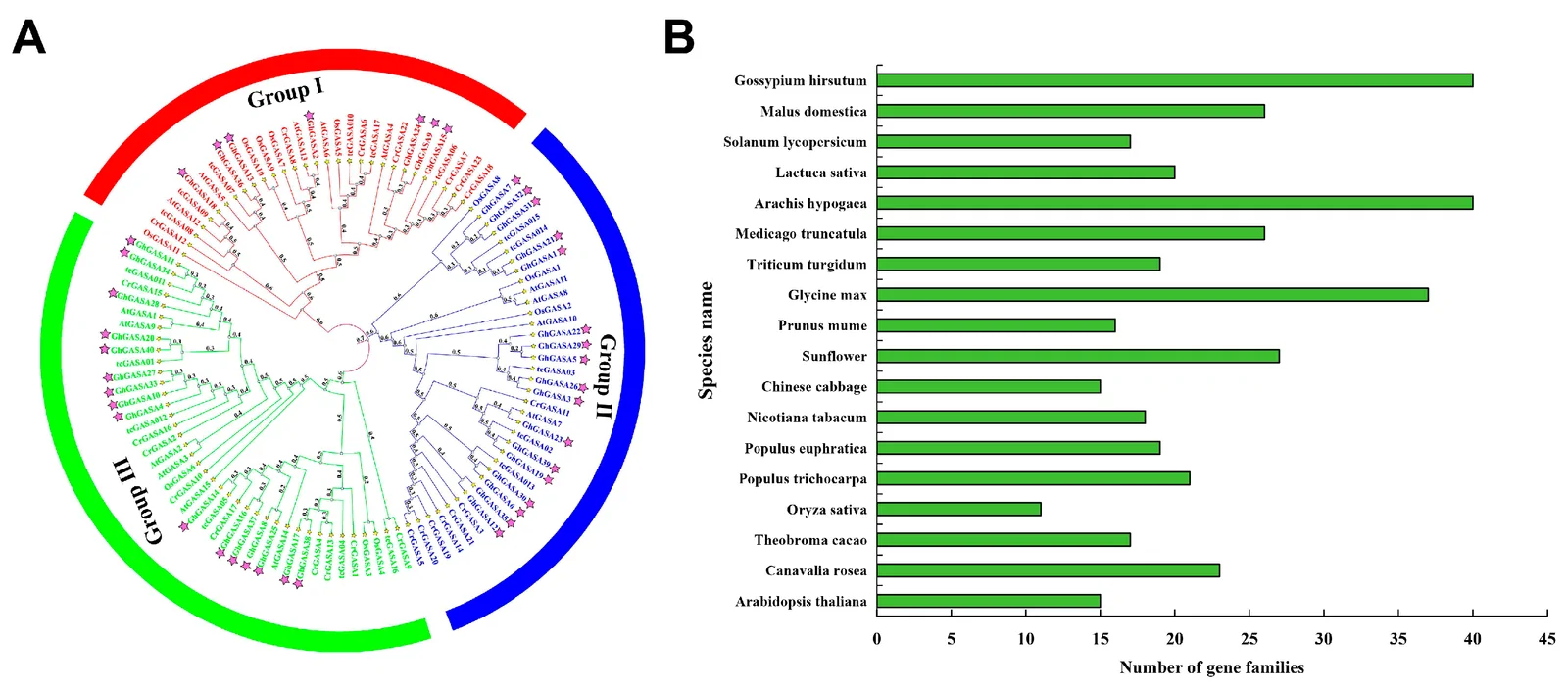
Phylogenetic tree of the *GhGASA* gene family in *G. hirsutum* and the number of *GhGASA* genes across multiple species. (**A**) Phylogenetic tree of the *GASA* gene family. *GASAs* are divided into three subclades based on branch color; the *GhGASA* gene family in *G. hirsutum* is marked with an asterisk. (**B**) Number of *GASA* family genes in 18 species. *Gossypium hirsutum*, *Arabidopsis thaliana*, *Canavalia rosea*, *Theobroma cacao*, *Oryza sativa*, *Populus trichocarpa*, *Populus euphratica*, *Nicotiana tabacum*, *Chinese cabbage*, *Sunflower*, *Prunus mume*, *Glycine max*, *Triticum turgidum*, *Medicago truncatula*, *Arachis hypogaea*, *Lactuca sativa*, *Solanum lycopersicum*, *Malus domestica*.

A phylogenetic tree was constructed based on 40 amino acid sequences from *G. hirsutum GhGASA* and 66 amino acid sequences from other species. Phylogenetic analysis divided the *GhGASA* gene family into three distinct branches with varying numbers of members: subfamily I contains 7 *GhGASA* family members, subfamily II contains 17, and subfamily III contains 16 (Figure 2A). The number of *GASA* gene family members varies among species; *G. hirsutum* and *Arachis hypogaea* have 40 members, while *Oryza sativa* has only 11 (Figure 2B).

**Figure 3:**
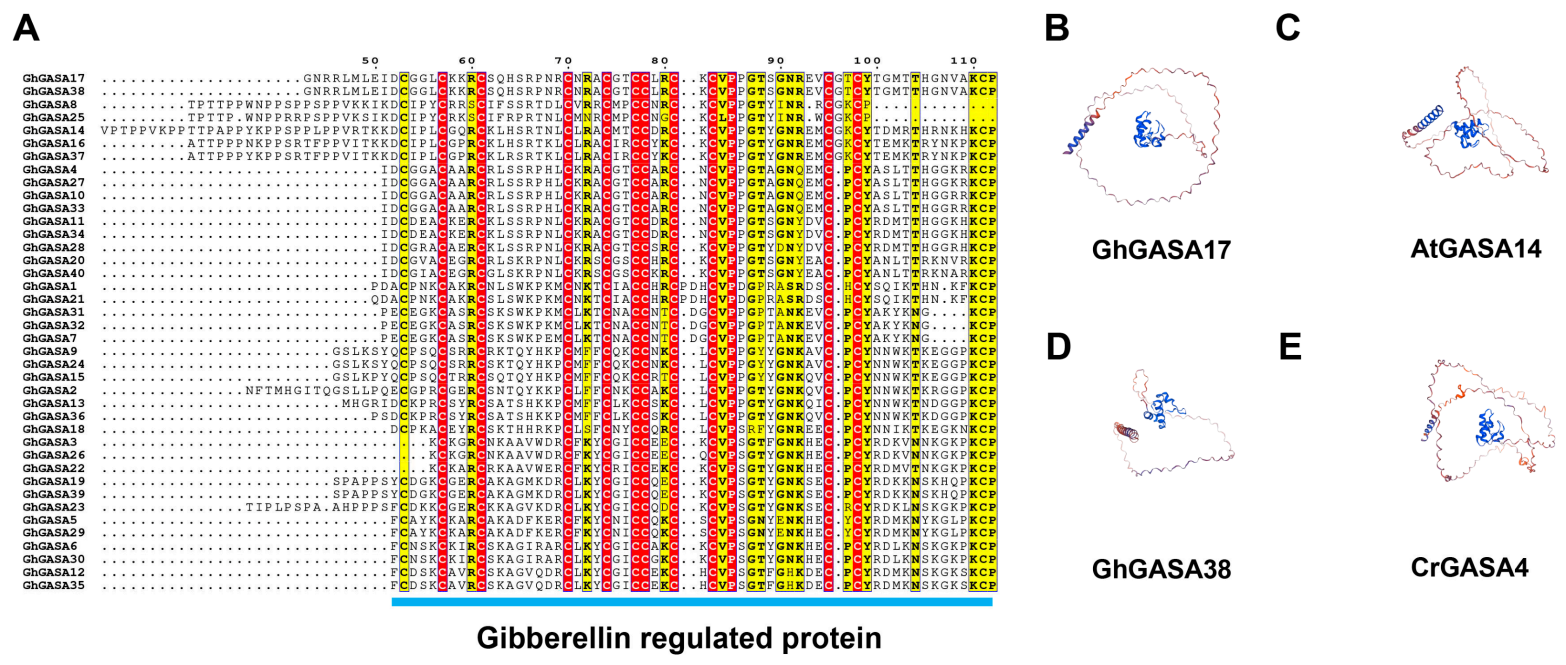
Amino acid sequence analysis of the *GhGASA* gene family and tertiary structures of *GASA* genes from three species. (**A**): Amino acid sequence alignment of the *GhGASA* gene family; the blue horizontal line indicates the conserved domain of the Gibberellin-regulated protein. In the alignment, red indicates identical amino acid residues, and yellow indicates conserved substitutions among the aligned sequences. (**B**–**E**): Tertiary structures of *GASA* genes from different species; (**B**): *GhGASA17*; (**C**): *AtGASA14*; (**D**): *GhGASA38*; (**E**): *CrGASA4*.

To further verify conservation, a CLUSTALX multiple sequence alignment was performed on GASA proteins from *G. hirsutum*. The results showed that all GASA proteins contain the typical conserved domain of gibberellin-regulated proteins, and GASA proteins from different species have similar tertiary structures (Figure 3). Although *GhGASA8/25* lacks one cysteine residue, it also contains the PF02704 domain and belongs to the *GASA* superfamily of proteins. In summary, the *GhGASA* gene family is highly conserved during evolution, and its structure shows similarity between *G. hirsutum* and other species.

### 3.3. Analysis of Conserved Motifs, Conserved Domains, and Gene Structures in the GhGASA Gene Family

**Figure 4:**
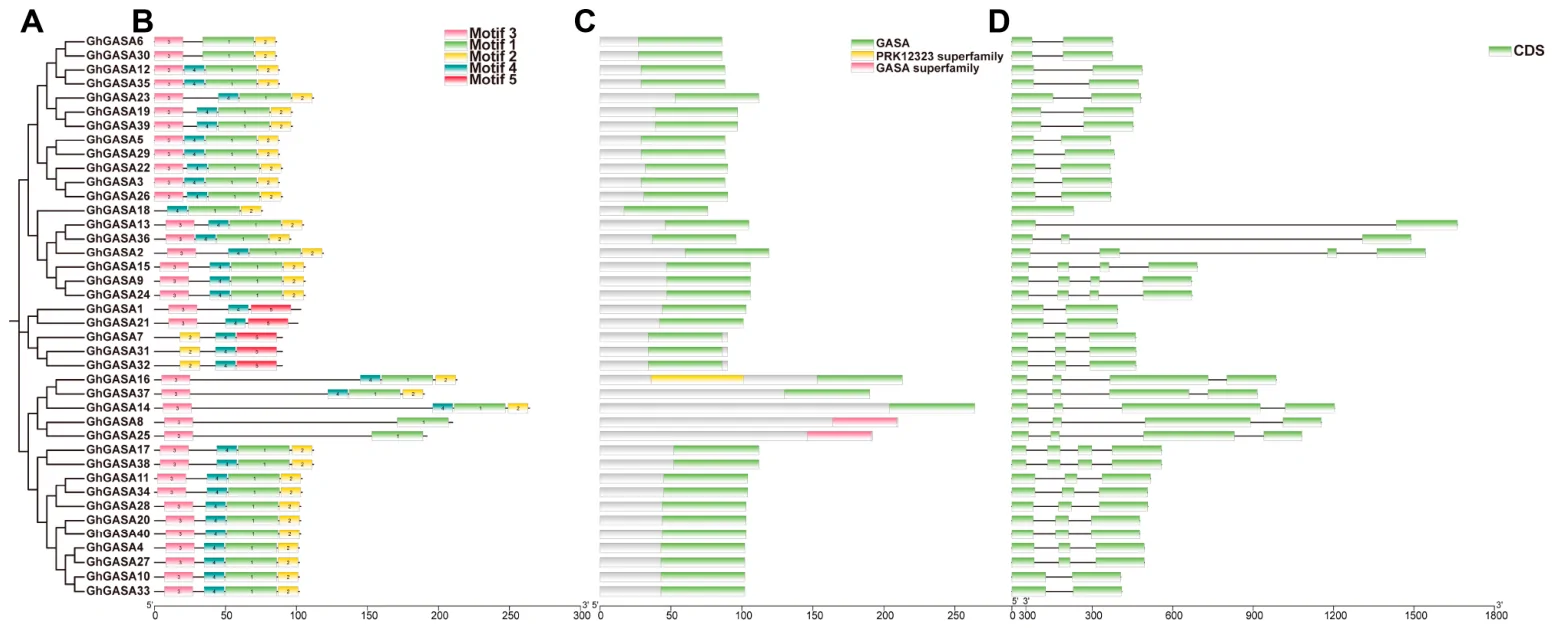
Analysis of conserved protein structures and gene structures in the *GhGASA* gene family of *G. hirsutum*. (**A**) Phylogenetic analysis of the *GhGASA* gene family. (**B**) Analysis of conserved protein motifs in the *GhGASA* gene family; different colors and numbers represent different conserved motifs. (**C**) Analysis of conserved protein domains in the *GhGASA* gene family; different colors represent different domains. (**D**) Analysis of the gene structure of the *GhGASA* gene family, where light green rectangles represent exons and black lines represent introns.

A multidimensional analysis of the *GhGASA* family was conducted by integrating phylogenetic trees, conserved motifs, protein domains, and gene structures (Supplementary Table S2, Figure 4). A total of five conserved motifs were identified among the 40 GhGASA proteins. Although the number and distribution of motifs varied among individual members, the family as a whole was highly conserved: all members contained at least two motifs, and the motif composition was consistent among members within the same clade. Domain analysis revealed that all proteins possess the GASA core domain, with *GhGASA16* additionally containing the PRK12323 domain. Regarding gene structure, 39 members contain 1–3 introns (*GhGASA18* has no introns), and the number of exons ranges from 1 to 4.

### 3.4. Collinearity Analysis of the GhGASA Gene Family

**Figure 5:**
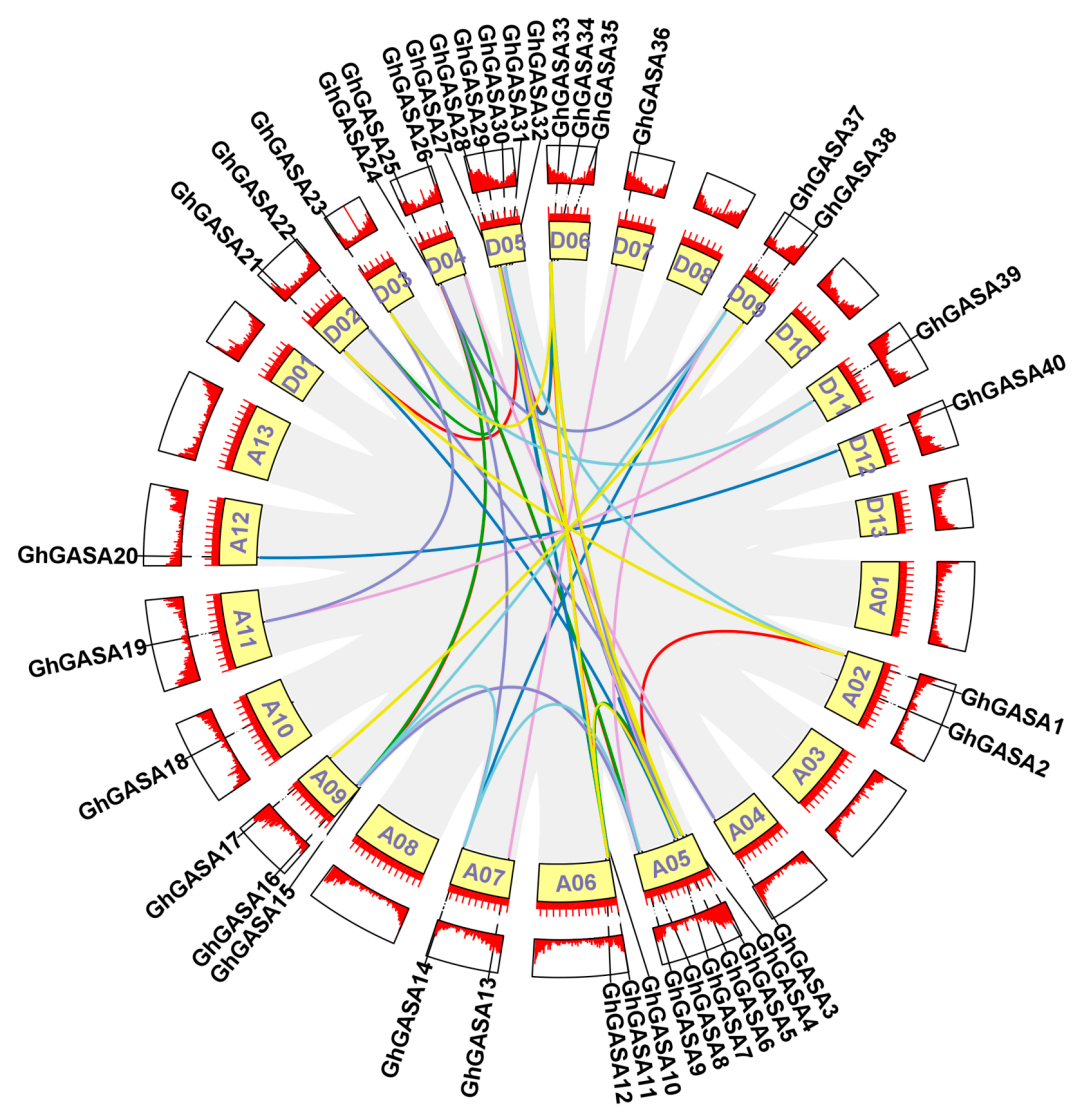
Analysis of intraspecific collinearity of *GhGASAs* in *G. hirsutum*. The light yellow fan-shaped rings represent *G. hirsutum* chromosomes of varying sizes; the gray lines in the background of the collinearity analysis represent genome-wide collinearity blocks, while the colored lines represent homologous pairs of *GhGASA* genes.

**Figure 6:**
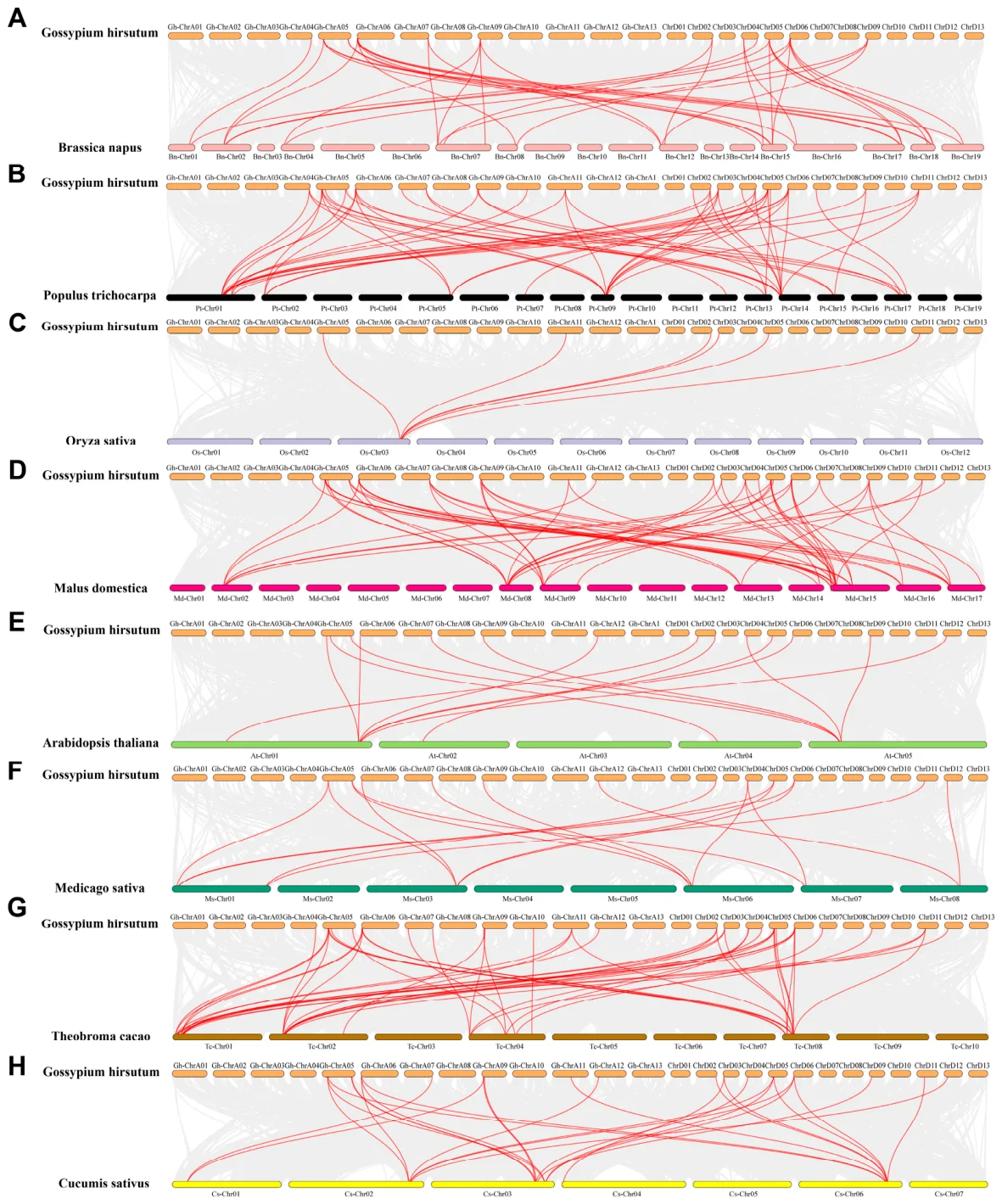
Analysis of interspecies collinearity of the *GhGASA* gene family in *G. hirsutum*. Collinearity analysis of the *GhGASA* gene family with 8 representative plant species ((**A**): *Brassica napus*, (**B**): *Populus trichocarpa*, (**C**): *Oryza sativa*, (**D**): *Malus domestica*, (**E**): *Arabidopsis thaliana*, (**F**): *Medicago sativa*, (**G**): *Theobroma cacao*, (**H**): *Cucumis sativus*). The gray lines in the background represent collinearity blocks between the *G. hirsutum* genome and those of other species, while the red lines indicate homologous *GASA* gene pairs between two species.

Intraspecific collinearity analysis identified 51 *GhGASA* fragment duplication events (Figure 5). To elucidate interspecies evolutionary relationships, collinearity analyses were performed between *G. hirsutum* and *Brassica napus*, *Populus trichocarpa*, *Oryza sativa*, *Malus domestica*, *Arabidopsis thaliana*, *Medicago sativa*, *Theobroma cacao*, and *Cucumis sativus*, yielding 38, 55, 6, 55, 14, 17, 49, and 27 homologous gene pairs, respectively (Figure 6A). Ka/Ks analysis revealed that in the *GhGASA* gene family, only two gene pairs (*GhGASA8* and *GhGASA25*, and *GhGASA10* and *GhGASA33*) had a Ka/Ks ratio greater than 1, while the Ka/Ks ratios for all other gene pairs were less than 1 (Supplementary Table S3).

### 3.5. Analysis of Cis-Acting Elements in the Promoters of the GhGASA Gene Family

**Figure 7:**
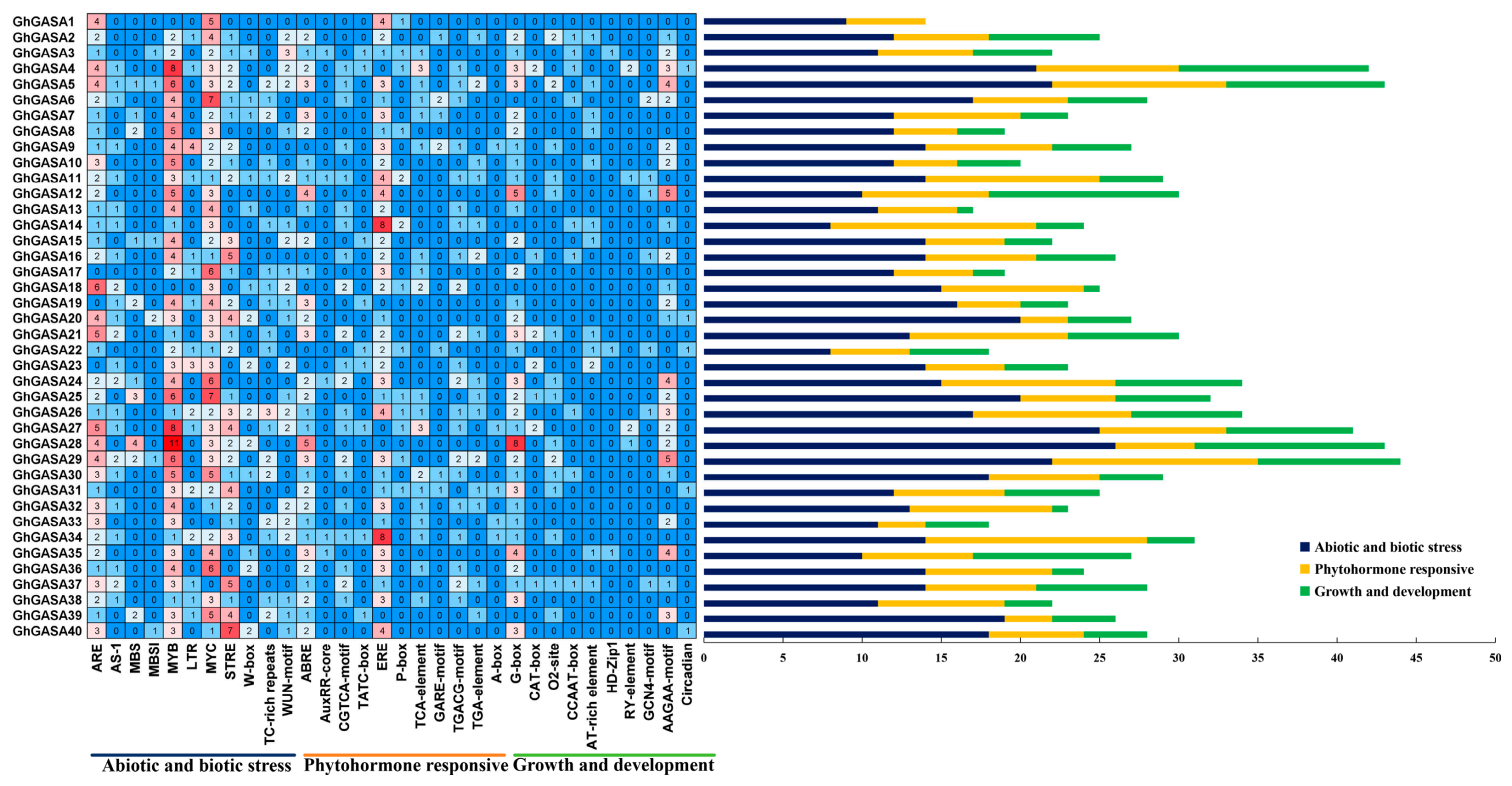
Analysis of cis-acting elements in the *GhGASA* gene family of *G. hirsutum*. The numbers in the heatmap on the left indicate the number of cis-acting elements for each gene. The different colors in the histogram on the right represent the number of cis-acting elements for each gene across three categories, classified by function as follows: Abiotic and biotic stress, Phytohormone responsive, and Growth and development.

To understand the expression patterns of the *GhGASA* gene family, cis-acting elements in the promoters of the *GhGASA* gene family were analyzed using the PlantCARE database (Figure 7). These cis-acting elements are involved in the growth and development of *G. hirsutum*, as well as in stress responses (including abiotic and biotic stresses) and plant hormone responses. Some *GhGASA* genes are enriched with stress-related cis-acting elements (such as MYB, MYC, and STRE), and these *GhGASA* genes (e.g., *GhGASA27/28*) may play a key role in responding to adverse environmental conditions. Furthermore, the promoters of the *GhGASA14/34* genes are enriched with ERE (Ethylene Response Element) cis-regulatory elements, suggesting they may be responsive to ethylene. The promoters of *GhGASA12/28/29* contain a large number of promoter elements associated with growth and development, such as the AAGAA motif and G-box.

### 3.6. Expression Patterns of the GhGASA Gene Family

**Figure 8:**
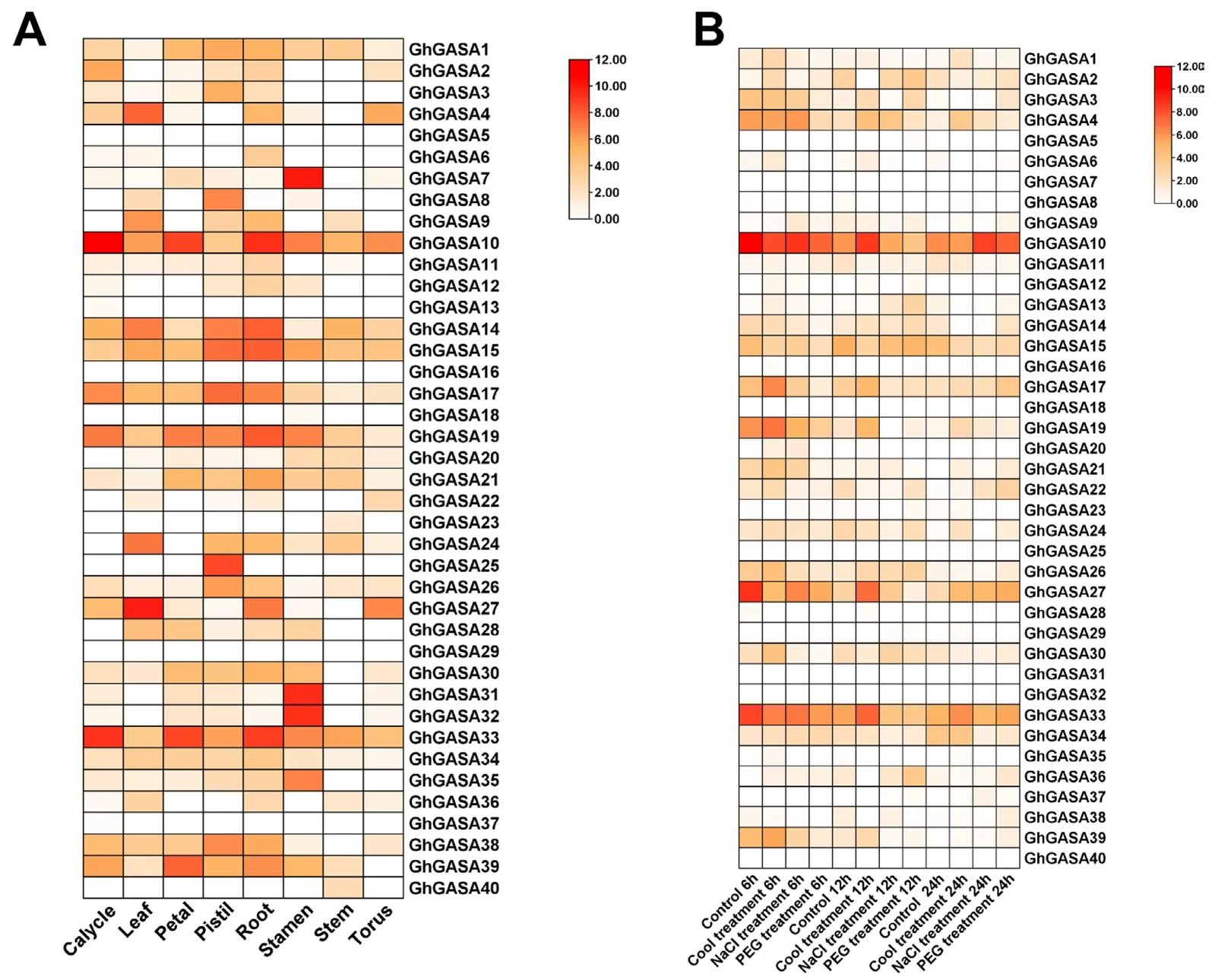
Transcription levels (in FPKM) of the *GhGASA* gene family in *G. hirsutum* across different tissues and under various abiotic stresses. (**A**) Expression levels of the *GhGASA* genes in eight different tissues, plotted based on transcriptomic data. (**B**) Heatmap showing *GhGASA* gene expression levels following cold stress, NaCl stress, and PEG-induced drought stress at three time points. The color bar on the right indicates the range between the maximum and minimum expression values in the heatmap. Red, orange, and white represent high, medium, and low expression levels, respectively.

To further elucidate the functions of the *GhGASA* gene family in cotton growth and development as well as in response to abiotic stress, this study visualized the expression profiles of *GhGASAs* in different tissues of *G. hirsutum* and under various abiotic stress conditions using heatmaps (Supplementary Table S4, Figure 8). The tissue expression heatmap shows that the vast majority of *GhGASAs* are expressed in the pistil and root, such as *GhGASA14*, *GhGASA15*, and *GhGASA17*; *GhGASA10*, however, maintains relatively high levels in all eight tissues, indicating that *GhGASA10* may play an important regulatory role in the overall growth and development of cotton. Stress expression heatmaps indicated that genes such as *GhGASA4* were significantly upregulated under salt stress, while *GhGASA17* and *GhGASA19* showed enhanced expression under cold stress. Under PEG-simulated drought stress, *GhGASA36* was significantly upregulated. These results reveal that members of this gene family are involved in the response processes to various abiotic stresses.

**Figure 9:**
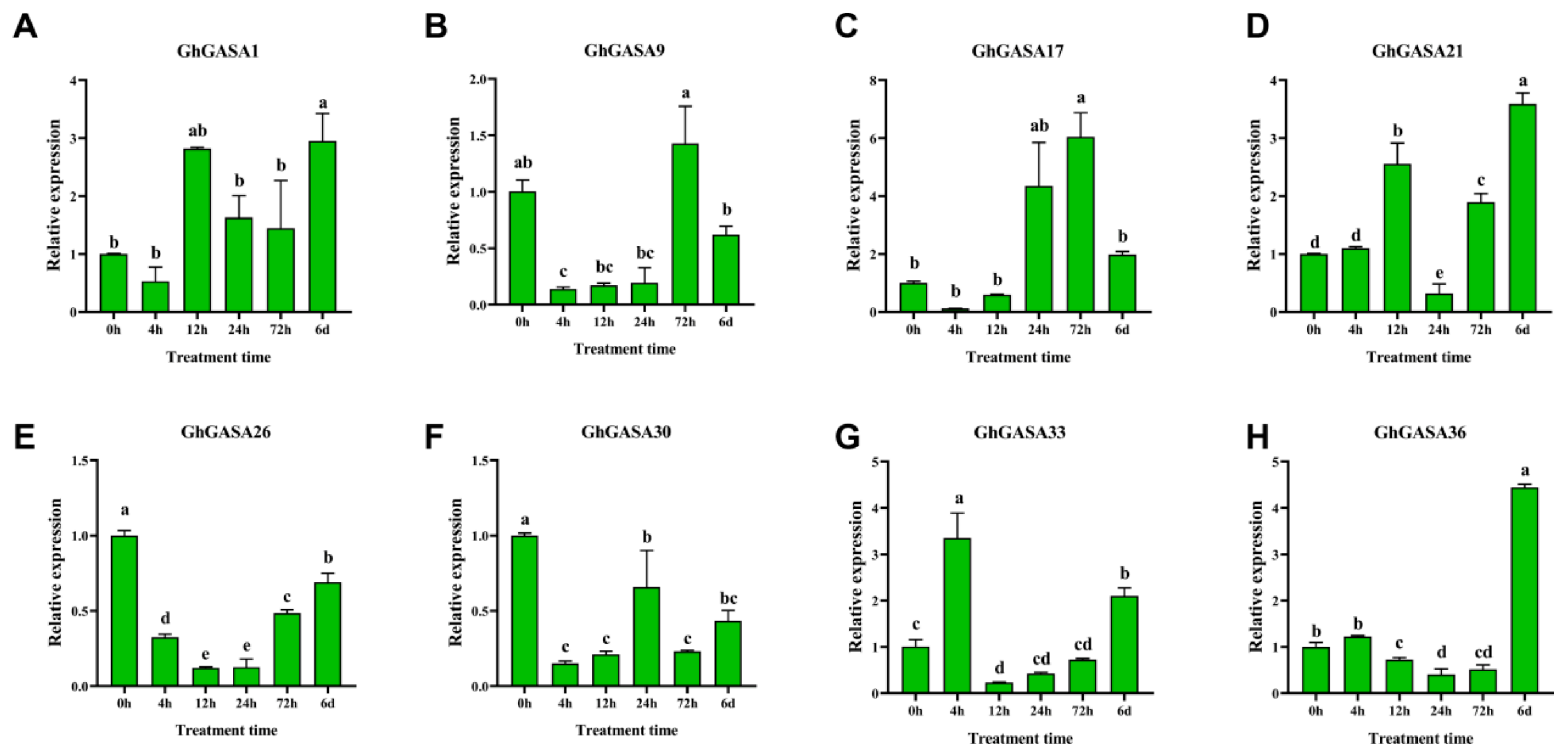
qRT-PCR analysis of *GhGASA* gene expression in leaves following *Verticillium wilt* infection. Panels (**A**–**H**) present the relative expression of *GhGASA1*, *GhGASA9*, *GhGASA17*, *GhGASA21*, *GhGASA26*, *GhGASA30*, *GhGASA33*, and *GhGASA36*, respectively. Data are shown as mean ± SD (*n* = 3). Columns with different letters are significantly different at *p* < 0.05 according to the Bonferroni test.

**Figure 10:**
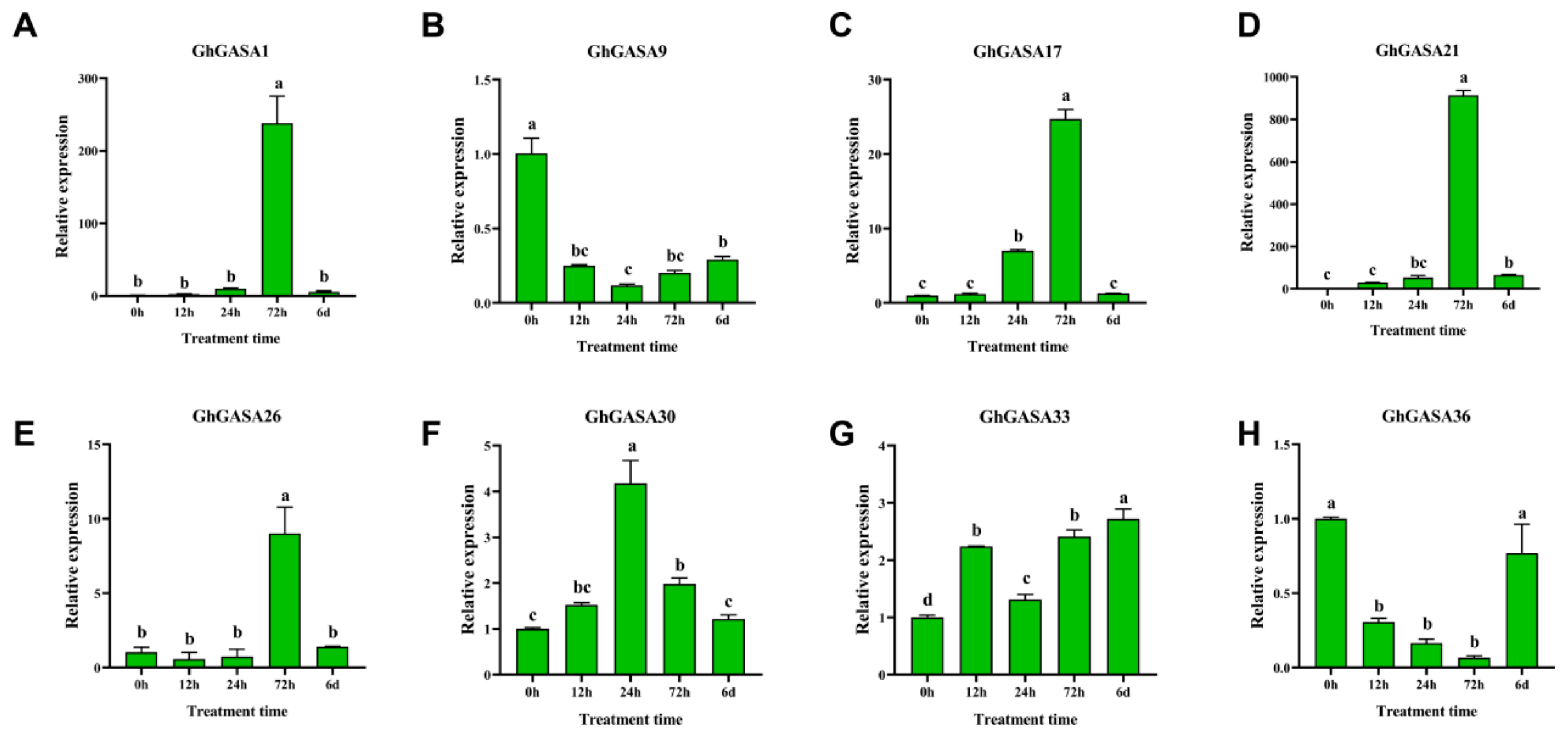
qRT-PCR analysis of *GhGASA* gene expression in stems following *Verticillium wilt* infection. Panels (**A**–**H**) present the relative expression of *GhGASA1*, *GhGASA9*, *GhGASA17*, *GhGASA21*, *GhGASA26*, *GhGASA30*, *GhGASA33*, and *GhGASA36*, respectively. Data are shown as mean ± SD (*n* = 3). Columns with different letters are significantly different at *p* < 0.05 according to the Bonferroni test.

**Figure 11:**
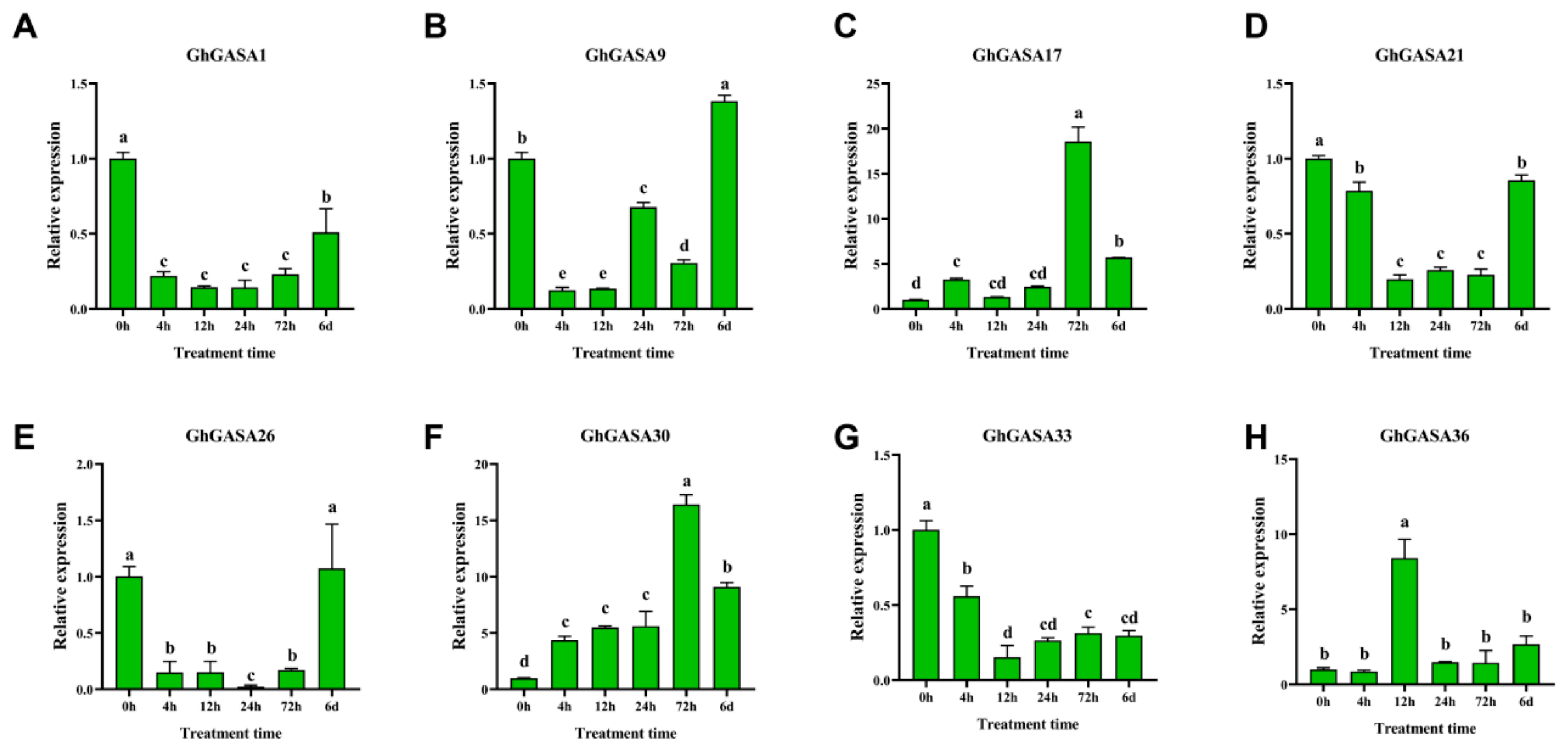
qRT-PCR analysis of *GhGASA* gene expression in roots following *Verticillium wilt* infection. Panels (**A**–**H**) present the relative expression of *GhGASA1*, *GhGASA9*, *GhGASA17*, *GhGASA21*, *GhGASA26*, *GhGASA30*, *GhGASA33*, and *GhGASA36*, respectively. Data are shown as mean ± SD (*n* = 3). Columns with different letters are significantly different at *p* < 0.05 according to the Bonferroni test.

qRT-PCR analysis revealed that the *GhGASA* gene family members exhibited distinct expression patterns in response to *Verticillium wilt* stress in *G. hirsutum*. The expression levels of *GhGASAs* were significantly higher in roots and stems than in leaves following stress treatment (Figure 9, Figure 10 and Figure 11). After *Verticillium wilt* inoculation, *GhGASA1/17/21/36* were up-regulated in leaves, while *GhGASA26* was downregulated. *GhGASA33* was initially upregulated in leaves but recovered to basal levels by 24 h post-inoculation. In stems, *GhGASA1/17/21/26/30/33* were upregulated with relatively high fold changes (those of *GhGASA1/17/21* all exceeded 20-fold), whereas *GhGASA9/36* were downregulated. In roots, *GhGASA9/17/30/36* were upregulated, while *GhGASA1/21/33* were downregulated. *GhGASA36* was upregulated at 12 h post-inoculation and then returned to basal levels, whereas *GhGASA26* was initially downregulated and recovered after 6 days.

## 4. Discussion

GASA proteins, also known as Snakin proteins, are cysteine-rich antimicrobial peptides that play crucial roles in plant innate immunity [48]. Their C-terminal GASA domain, characterized by 12 conserved cysteine residues, is essential for maintaining structural stability and biological function [26,49]. Mechanistically, GASA proteins contribute to disease resistance through multiple pathways, including direct antimicrobial activity, as demonstrated by the durum wheat TdGASA1 protein, which exhibits antagonistic activity against various fungal pathogens [50]; modulation of phytohormone signaling, particularly the salicylic acid (SA) pathway, as shown by *AtGASA1* and *AtGASA4* in *Arabidopsis* [29,51]; and regulation of ROS homeostasis, a key early event in plant innate immunity [34]. This multi-mechanistic framework suggests that the *GhGASA* genes identified in our study may similarly function through a combination of direct antimicrobial effects and hormone-mediated signaling to coordinate cotton defense against *Verticillium wilt*.

In recent years, the role of GASA proteins in plant immunity has attracted increasing attention. These proteins participate in disease resistance regulation through at least three mechanisms: direct antimicrobial peptide activity to inhibit pathogen growth; indirect modulation of defense responses via plant hormone signaling pathways (particularly the SA, JA, and GA pathways); and mediation of early immune signaling through ROS homeostasis [33,34,50]. Our finding that multiple *GhGASA* members are significantly upregulated in roots, stems, and leaves following *V. dahliae* infection is consistent with this multi-mechanistic framework, suggesting that *GhGASAs* may similarly contribute to cotton defense through coordinated actions at both the direct antimicrobial and signaling levels (Figure 9, Figure 10 and Figure 11). From an evolutionary perspective, the expansion of the *GhGASA* family from diploid progenitors to allopolyploid *G. hirsutum* generally follows an additive trend, reflecting the AADD genomic composition of upland cotton [52]. Notably, segmental duplication, rather than tandem duplication, was identified as the primary driver of this expansion (Figure 1 and Figure 5). This mode of duplication may have important functional implications: segmental duplication preserves large genomic contexts and regulatory landscapes, potentially allowing duplicated *GhGASA* copies to acquire distinct expression patterns and subfunctionalize in response to different stresses [30,32]. Such evolutionary flexibility may have enabled certain *GhGASA* members, including *GhGASA17*, to evolve specialized roles in vascular pathogen defense—a hypothesis supported by their robust transcriptional induction upon *Verticillium wilt* infection. At the protein structural level, GhGASA protein sequences range in length from 76 to 264 amino acids, with molecular weights generally less than 30 kDa; all members contain the typical Gibberellin-regulated protein domain (PF02704). This characteristic fully aligns with the molecular properties of GASA proteins as small-molecule CRPs (Supplementary Table S1, Figure 4). Tertiary structure predictions indicate that representative proteins such as GhGASA17 and GhGASA38 share highly similar spatial conformations with *Arabidopsis* AtGASA14 and cocoa CrGASA4, suggesting that the folding pattern of this protein family is highly conserved across angiosperms (Figure 3B–E). Multiple sequence alignments reveal that the positions of the 12 cysteine residues within the *GASA* core domain are highly conserved across all members. The disulfide bond network formed by these 12 cysteines is considered critical for maintaining the three-dimensional structure and biological activity of GASA proteins (Figure 3A). It is precisely this highly conserved molecular framework that provides the structural basis for GASA proteins to perform similar functions across different species.

The preferential expression of *GhGASA* genes in roots and pistils is functionally relevant to their potential roles in development and defense. The high expression in roots, the primary infection site of *Verticillium dahliae*, closely parallels the specific expression of potato Snakin-3 in root and stem vascular tissues, suggesting that GASA proteins may contribute to defense against soil-borne pathogens through local accumulation at vascular infection sites [48]. In addition to biotic stress, many *GhGASA* members also respond to abiotic stresses including salinity, drought, and cold (Figure 8B). This multi-stress responsiveness is consistent with the enrichment of MYB, MYC, and STRE cis-elements in their promoters, implying that *GhGASA* genes may function as general stress-responsive nodes (Figure 7). Notably, *GhGASA17*-which showed the strongest induction under *Verticillium wilt*-also responded to cold stress, raising the possibility that this particular member serves as a convergence point for both biotic and abiotic stress signaling. This feature is consistent with the emerging view of GASA proteins as integrators of multiple hormonal and environmental signals, and suggests that specific *GhGASA* members could be promising targets for improving broad-spectrum stress tolerance in cotton [26,36].

The strong and tissue-specific induction of *GhGASA17* under *Verticillium wilt* infection warrants particular attention. Its promoter is enriched with MYC and ERE motifs, implicating this gene in both jasmonate and ethylene signaling pathways—both of which are central to cotton defense against *V. dahliae* [18,20]. The ethylene pathway is particularly relevant, as ethylene signaling has been shown to positively regulate cotton resistance to *Verticillium wilt* by activating defense-related gene expression and promoting phytoalexin accumulation [20]. The presence of ERE elements in the *GhGASA17* promoter raises the possibility that this gene may function as a downstream effector of ethylene-mediated defense. Interestingly, *GhGASA17* also responded to cold stress, suggesting that its regulatory network may integrate signals from multiple stress pathways (Figure 8B). We hypothesize that upon *V. dahliae* infection, ethylene signaling activates *GhGASA17* expression, which in turn may contribute to defense through its GASA-encoded antimicrobial peptide activity or through modulation of downstream hormone signaling. This working model is consistent with the established role of GASA proteins as hormonal signaling integrators and provides a testable hypothesis for future functional studies [26].

Although Qiao et al. (2021) previously conducted a genome-wide identification of the *GASA* family across five cotton species, their study primarily focused on basic phylogenetic distribution and abiotic stress responses, leaving the involvement of this family in biotic stress particularly *Verticillium wilt* entirely unexplored [52]. Here, we address this critical gap by systematically investigating the transcriptional dynamics of the *GhGASA* family under *Verticillium dahliae* infection in *G. hirsutum*. Our study provides several distinct advances, we updated the gene inventory to 40 members using the latest *TM-1 v2.1* genome; we revealed fine-tuned tissue-specific expression patterns across roots, stems, and leaves over a detailed 6-time-course infection series, identifying *GhGASA1/17/21* as strongly upregulated candidates (over 20-fold in stems); and we pinpointed *GhGASA17* as a key candidate gene, whose promoter is enriched with ethylene-responsive (ERE) and MYC elements, suggesting its potential integration into the ethylene-mediated defense pathway against *Verticillium wilt*. While direct transgenic validation of *GhGASA17* remains a priority for future work, the core novelty of this study lies in its explicit biological focus addressing *Verticillium wilt* resistance rather than general abiotic stress tolerance and its delivery of actionable genetic resources for cotton molecular breeding. Thus, this research not only complements prior genomic surveys but also establishes a functional framework for leveraging *GhGASA* genes in enhancing cotton resistance to vascular diseases. It should be noted that the present study focuses on transcriptomic profiling and candidate gene mining; phenotypic evaluation and fungal biomass quantification will be incorporated in our ongoing functional validation studies.

## 5. Conclusions

Through genome-wide analysis, this study identified 40 members of the *GhGASA* family in *G. hirsutum*. Members of the *GhGASA* gene family were classified into three subfamilies, with genes within each subfamily exhibiting highly conserved motifs, domains, gene structures, and evolutionary relationships. The expansion of the *GhGASA* gene family in *G. hirsutum* was driven by four tandem duplication and 51 fragment duplication events. Inter-species synteny analysis revealed strong synteny with dicotyledonous woody plant species, highlighting potential evolutionary conservation. Analysis of promoter cis-acting elements revealed the potential roles of *GhGASA* genes in stress response and hormone signaling regulation. Transcriptomic analysis indicated that the *GhGASA* genes are highly expressed in roots and pistils and respond to abiotic stresses such as salinity, drought, and low temperature. Transcriptomic and qRT-PCR analyses following *Verticillium wilt* infection revealed that several candidate genes were significantly upregulated in roots, stems, and leaves after infection. These genes may be associated with enhanced *Verticillium wilt* resistance in cotton; however, direct functional analyses (such as transgenic studies) are needed to confirm their roles. Based on the above results, genes such as *GhGASA17* were identified as candidate genes worthy of further functional characterization in the *Verticillium wilt* resistance response in upland cotton. This study provides a theoretical basis for further elucidating the role of the *GhGASA* gene in the disease resistance regulatory network of cotton and offers new genetic resources for molecular breeding aimed at enhancing cotton resistance to *Verticillium wilt*.

## Data Availability

All data supporting the conclusions of this paper are provided in the article and Supplementary Materials. Genome sequence data for all species are available in the Phytozome database (https://phytozome-next.jgi.doe.gov/ (accessed on 17 August 2026)) and COTTONOMICS database (http://cotton.zju.edu.cn/index.htm (accessed on 17 August 2026)). Publicly available RNA-seq data are available on the COTTONOMICS database.

## Supplementary Materials

The following supporting information can be downloaded at: https://www.mdpi.com/article/10.3390/biology15161434/s1, Table S1. List of *Gossypium_hirsutum GhGASA* genes; Table S2. Sequence and SeqLogo of Motif1–5; Table S3. Duplications of *GhGASA* genes in *Gossypium hirsutum L.*; Table S4. Expression of *GhGASA* genes; Table S5. Primers used for RT-qPCR in the article.
